# Low incidence of HIV infection and decreasing incidence of sexually transmitted infections among PrEP users in 2020 in Germany

**DOI:** 10.1007/s15010-022-01919-3

**Published:** 2022-09-27

**Authors:** Daniel Schmidt, Christian Kollan, Barbara Bartmeyer, Viviane Bremer, Tim Schikowski, Martin Friebe, Sven Schellberg, Stefan Scholten, Markus Bickel, Nikola Hanhoff, Robin Rüsenberg, Knud Schewe, The NEPOS study group, Heribert Knechten, Petra Panstruga, Axel Baumgarten, Bianca Reisenweber, Heribert Hillenbrand, Kai Zucker, Siegfried Köppe, Marc Da Silva Ribeiro, Ivanka Krznaric, Denis Pitan, Christian Lieb, Hussen Yasin, Ingo Ochlast, Irina Shnurenko, Michael Rausch, Svetlana Krasov, Sven Schellberg, Peter Stueber, Dietmar Schranz, Michael Rittweger, Hubert Schulbin, Adam Smiley, Kevin Ummard-Berger, Andreas Berger, Johannes Lenz, Chistoph Boesecke, Martha Oberschlep, Susann Koch, Mareen Monsees, Thomas Heuchel, Manuela Richter, Andreas Bellmunt-Zschäpe, Jutta Hahn, Andreas Jenke, Stefan Pursche, Stefan Mauss, Florian Berger, Marcus Bickel, Monja Rößler, Anette Haberl, Peter Schott, Pavel Khaykin, Michaela Bracone, Susanne Usadel, Tina Mattmüller, Georg Friese, Ulrike Kratz, Sandra Hertling, Dirk Berzow, Knud Schewe, Constantin Rickassel, Hans Heiken, Ansgar Rieke, Petra Becker, Katja Römer, Stefan Scholten, Andreas Roder, Christoph Wyen, Kerstin Lammersmann, Ines Ruck, Burkhard Schappert, Ulrich Kastenbauer, Ramona Pauli, Oskar Mikazans, Farhad Schabaz, Christoph Spinner, Stefanie Gladis, Stefan Christensen, Sabine Offermann, Robert Baumann, Niels Schübel, Anja Lüssenheide, Franz Audebert, Elisabeta Sepsy, Beatrice Gospodinov, Aynur Bulut, Patrick Beck, Jennifer Weida, Georg Härter, Petra Schütz, Thomas Seidel, Sabine Mauruschat, Steve Rößler

**Affiliations:** 1 Department of Infectious Disease Epidemiology, Robert Koch Institute, Berlin, Germany; 2 Novopraxis, Berlin, Germany; 3 Praxis Hohenstaufenring, Cologne, Germany; 4 Infektiologikum, Frankfurt, Germany; 5 German Association of Physicians in HIV-Care (dagnä e.V.), Berlin, Germany

**Keywords:** Human immunodeficiency virus (HIV), HIV pre-exposure prophylaxis (PrEP), Sexually transmitted infections (STI), Germany, Health insurance coverage

## Abstract

**Introduction:**

Objectives of this study, as part of a nation-wide HIV pre-exposure prophylaxis (PrEP) evaluation project, were to determine the incidence of infections with HIV, chlamydia, gonorrhea, syphilis, hepatitis A/B/C in persons using PrEP, and to describe the health care funded PrEP use in Germany. Additionally, factors associated with chlamydia/gonorrhea and syphilis infections were assessed.

**Methods:**

Anonymous data of PrEP users were collected at 47 HIV-specialty centers from 09/2019–12/2020. Incidence rates were calculated per 100 person years (py). Using longitudinal mixed models, we analyzed risk factors associated with sexually transmitted infections (STIs).

**Results:**

4620 PrEP users were included: 99.2% male, median age 38 years (IQR 32–45), 98.6% men who have sex with men (MSM). The median duration of PrEP exposure was 451 days (IQR 357–488), totaling 5132 py. Four HIV infections were diagnosed, incidence rate 0,078/100py (95% CI 0.029–0.208). For two, suboptimal adherence was reported and in the third case, suboptimal adherence and resistance to emtricitabine were observed. One infection was likely acquired before PrEP start. Incidence rates were 21.6/100py for chlamydia, 23.7/100py for gonorrhea, 10.1/100py for syphilis and 55.4/100py for any STI and decreased significantly during the observation period. 65.5% of syphilis, 55.6% of chlamydia and 50.1% of gonorrhea cases were detected by screening of asymptomatic individuals. In a multivariable analysis among MSM younger age, PrEP start before health insurance coverage and daily PrEP were associated with greater risk for chlamydia/gonorrhea. Symptom triggered testing and a history of STI were associated with a higher risk for chlamydia/gonorrhea and syphilis. A significantly lower risk for chlamydia/gonorrhea and syphilis was found for observations during the COVID-19 pandemic period.

**Conclusions:**

We found that HIV-PrEP is almost exclusively used by MSM in Germany. A very low incidence of HIV infection and decreasing incidence rates of STIs were found in this cohort of PrEP users. The results were influenced by the SARS-CoV-2 pandemic. Rollout of PrEP covered by health insurance should be continued to prevent HIV infections. Increased PrEP availability to people at risk of HIV infection through the elimination of barriers requires further attention. Investigation and monitoring with a longer follow-up would be of value.

**Supplementary Information:**

The online version contains supplementary material available at 10.1007/s15010-022-01919-3.

## Introduction

Infection with the human immunodeficiency virus (HIV) remains globally endemic with 1.5 million new infections annually despite continued advancements in its management, treatment, and prophylaxis [1]. In Germany, HIV transmission remains an issue despite prophylactic measures, and a knowledgeable men who have sex with men (MSM) population which is the largest affected group. Of the estimated 91 400 people living with HIV in Germany at the end of 2020, an estimated 90% (~ 81 900) were diagnosed with HIV, 97% (~ 79 300) were receiving antiretroviral treatment and 96% (~ 76 500) were virally suppressed [2, 3]. Whereas prevention historically focused on condom use and safe injection practices, the approval of the antiviral agents tenofovir disoproxil fumarate and emtricitabine (TDF/FTC) for HIV pre-exposure prophylaxis (PrEP) has added a valuable tool for prevention in certain populations. Efficacy has been reported as high as 95% in MSM [4]. Unfortunately, similar results were not observed in women [5]. Since the efficacy of PrEP is highly correlated with adherence, an evaluation in the routine clinical care setting would provide further information on PrEP use and outcomes [6].

Although the use of PrEP significantly reduces the risk of HIV transmission, it may change sexual behavior resulting in an elevated risk for the transmission of other sexually transmitted infections (STIs) and even HIV [7–9]. On the other hand, PrEP access under medical supervision offers an opportunity for counselling, regular STI screening and appropriate treatment [7]. Earlier diagnosis of HIV infection and STIs and prompt treatment in turn may limit onward transmission.

PrEP was approved in Germany in 2016 and available initially on a self-paying basis [10]. As a result, it was frequently obtained from informal sources outside of the health care system and hence without adequate testing for HIV and other STIs, counselling and monitoring of side effects. Since September 2019, PrEP and its care by a certified and (HIV-) specialized physician is covered by the statutory health care system in Germany [11]. A previous analysis of PrEP use in Germany was based on prescription data and surveys among MSM and estimated a total of 15 600 to 21 600 PrEP users by the end of June 2020 [12]. However, further information on the characteristics of PrEP users, mode of PrEP use and incidences of HIV and other STI over time is required.

The uptake and outcome of PrEP use after introduction of coverage by the German statutory health insurance as well as any effects on the incidence of STIs were evaluated through a nation-wide study in Germany (“EvE-PrEP”) [13–18]. The evaluation consisted of several independent studies. The objectives of the NEPOS (National Evaluation of PrEP Outcomes and STIs) study, as part of the EvE-PrEP project, were to determine the incidence of infections with HIV, chlamydia, gonorrhea, syphilis, hepatitis A/B/C in persons using PrEP, and to describe the health care funded PrEP use in Germany. Additionally, factors associated with infections with chlamydia/gonorrhea and syphilis were assessed.

## Methods

### Study population and data collection

The NEPOS study was conducted by the Robert Koch Institute (RKI) in collaboration with the German association of physicians in HIV care (dagnä e.V.). All HIV-specialty care centers were invited to participate. The study was a retrospective longitudinal analysis of randomly selected PrEP users. The maximal number of PrEP users that could be documented in the study was set at 5000 for budgetary reasons. To ensure that the study sample is representative for the German PrEP using population, the centers were allocated a defined quota on the basis of their PrEP using population in reference to that of all participating centers. The randomization process at each center was performed at the discretion of the participating center.

Data were collected anonymously using an electronic reporting form in the first quarter of 2021 retrospectively for the time-period of September 2019 through December 2020. Date of individual PrEP initiation before September 2019 was documented if persons started PrEP before the observation period. STI with chlamydia, gonorrhea or syphilis 6 months prior to PrEP initiation were also documented (patient file or self-reported).

The study centers entered the data into the electronic reporting form from routine patient files. Data included patient characteristics (age, gender), PrEP use (indication, starting date, daily vs. intermittent/on-demand use as well as any interruption or discontinuation dates and reasons), testing for and potential diagnosis of HIV, hepatitis and STI, as well as hepatitis A and B antibody status. Duration of PrEP use was calculated as the number of days between PrEP start and end of follow-up, the latter defined by either PrEP discontinuation or the end of the study, 31/12/2020. Aside from HIV infection, STI included chlamydia, gonorrhea and syphilis. Newly diagnosed viral hepatitis included A, B and C. The observation period was divided into five time intervals: First September through December 2019, then four quarters in 2020 (2020q1–2020q4). Gonorrhea, chlamydia and syphilis were counted for each time interval, HIV and hepatitis with a date of diagnosis.

### STI, HIV and hepatitis incidence rates

Testing for HIV and STIs was performed in all centers as recommended by the German/Austrian PrEP guidelines [19]. In accordance with Ong et al., STI diagnosis within 90 days of PrEP initiation was defined as baseline prevalence [20]. Incidence rates were determined for STIs occurring while on PrEP and calculated by dividing the number of STI cases by the duration of PrEP exposure per time interval as per 100 person years (py). For incidence rates of infections with hepatitis and HIV, the duration of PrEP exposure in the observation period to the date of diagnosis was calculated as person-time. More than one positive test for a certain STI within one time interval was counted as one result. This procedure was chosen, because STIs were reported as the number of infections per time interval without the precise date of diagnosis. Additionally, because these events were rare, a sensitivity analysis showed a negligible effect of multiple infections with the same STI within a single time interval on STI incidence rates. It was predefined that PrEP was only considered interrupted or discontinued if stopped for longer than 4 weeks. As an additional indicator for either intermittent/on-demand PrEP use or suboptimal adherence to a documented daily PrEP, PrEP pill coverage was calculated using the number of pills prescribed divided by the number of days on PrEP. In case of a surplus of pills the number of pills were adjusted to the total duration of PrEP exposure to calculate the average PrEP pill coverage, assuming that for daily PrEP a maximum of one pill per day is taken (e.g. a prescription of 5 × 90 = 450 pills over a duration of 365 days results in a PrEP pill coverage of 1.23 which for the calculation of the average PrEP pill coverage was adjusted to 1.0).

### Statistical analysis

Univariable and multivariable longitudinal multilevel mixed-effects logistic regression corrected for the study centers as random intercept were used to analyze risk factors associated with infections with chlamydia/gonorrhea and syphilis. The analysis was performed among all MSM with/without other risk factors being tested in the respective time interval adjusted for age, PrEP use (intermittent/on-demand vs. daily), PrEP interruptions, PrEP discontinuations, PrEP duration, PrEP start before statutory health insurance coverage, infections with Hepatitis or HIV, other STI than the one analyzed, STI history (any STI yes/no) in the past 6 months before PrEP start, the number of STI tests, observation before (2019q4, 2020q1) or within (2020q2, 2020q3, 2020q4) the COVID-19 pandemic period.

### Sensitivity analysis of loss to follow-up

In a sensitivity analysis, we analyzed persons without any event related to PrEP use (testing, diagnosis, treatment of STI, HIV, Hepatitis, or PrEP prescriptions) for at least two quarters before their last event to calculate a ”loss to follow-up” rate for those without documentation of PrEP discontinuation or interruption.

Data were collected anonymously using an electronic reporting form programmed in C+ + . Data were stored and the dataset prepared using a MS-SQL server. Statistical analysis was performed using STATA Version 17. Figures were produced using Gfk RegioGraph and Microsoft Excel (Office 2019).

## Results

**Fig. 1:**
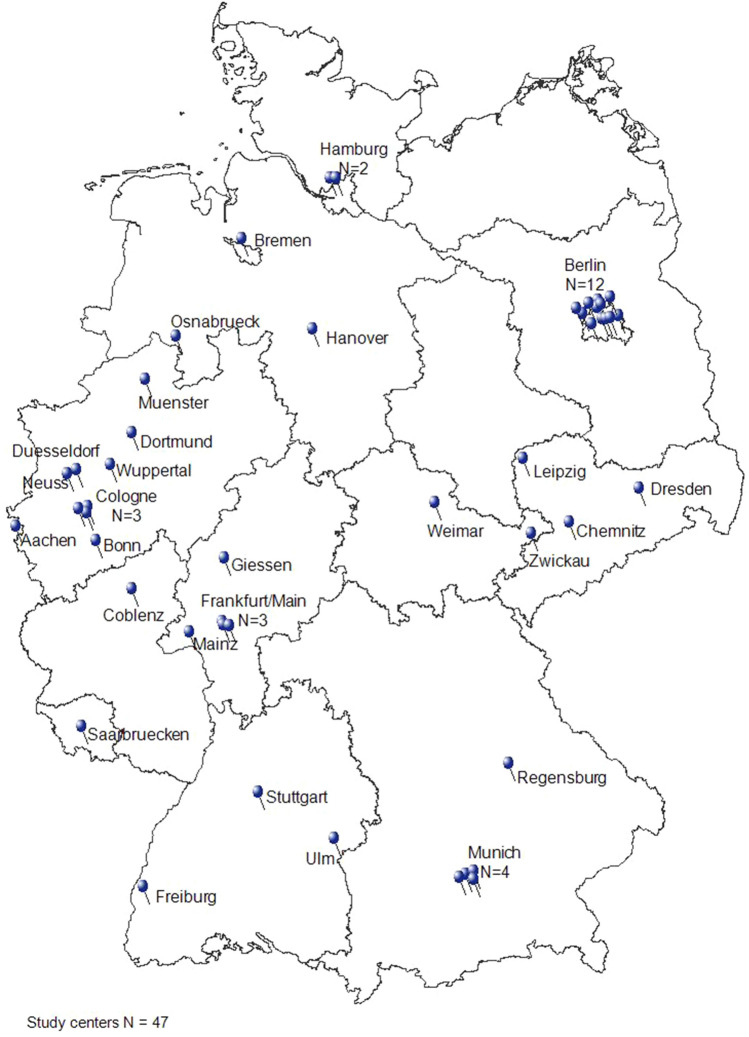
Distribution of participating study centers in Germany

Forty-seven German HIV-specialty care centers participated in the study and provided representation of PrEP use as a whole from both a geographical and a diversity perspective (Fig. 1).

4620 PrEP users were randomly selected to be documented in this study. Nearly all (99.2%) were male, median age was 38 years (IQR 32–45), 98.6% were MSM of whom 10.6% had additional risk factors. Of the 39 PrEP users who were not male, 17 were female, 16 transgender and four non-binary gender. Characteristics are shown in Table 1. The majority of cases (*n* = 1638, 35.5%) were documented in Berlin, which also reflects the pre-dominant location of current PrEP use.

**Table 1:** Characteristics of the study population

Total study population 4620	*N* (%)
Age (years)	
16–19	3 (0.1%)
20–29	715 (15.5%)
30–39	1864 (40.3%)
40–49	1233 (26.7%)
50–59	669 (14.5%)
≥ 60	136 (2.9%)
Gender	
Male	4581 (99.2%)
Female	17 (0.4%)
Diverse	4 (0.1%)
Trans (m- > f)	12 (0.3%)
Trans (f- > m)	6 (0.1%)
PrEP indication	
MSM	4065 (88.0%)
MSM and other high risk contacts	294 (6.4%)
MSM and HIV-discordant couple	142 (3.1%)
Other high-risk contacts	33 (0.7%)
MSM and IDU	25 (0.5%)
HIV-discordant couple	23 (0.5%)
IDU	1 (0.0%)
Other combinations	33 (0.7%)
Missing	4 (0.1%)
PrEP type of administration	
Daily	3737 (80.9%)
On Demand	874 (18.9%)
Missing	9 (0.2%)
PrEP start prior to 01/09/2019	
Yes	2466 (53.4%)
No	2106 (45.6%)
Unknown/missing	48 (1.0%)
PrEP interruption	
No	4159 (90.0%)
Yes	462 (10.0%)
PrEP discontinuation	
No	4012 (86.8%)
Yes	608 (13.2%)
Calculated PrEP pill coverage—PrEP prescriptions/time for all participants	
< 0.75	1069 (23.1%)
0.75–1.24	3184 (68.9%)
≥ 1.25	341 (7.9%)
Missing	26 (0.6%)
Hepatitis A immunity	
Yes	3358 (72.7%)
Incomplete	201 (4.4%)
No	299 (6.5%)
Not determined/missing	762 (16.5%)
Hepatitis B immunity	
Yes	3664 (79.3%)
Incomplete	336 (7.3%)
No	329 (7.1%)
Not determined/missing	291 (6.3%)
STI in the 6 months prior to PrEP start	
Chlamydia	231 (5.0%)
Gonorrhea	261 (5.6%)
Syphilis	234 (5.1%)
Multiple STI before PrEP start	196 (4.2%)
No STI in the 6 months prior to PrEP start	2777 (60.1%)
Unknown/missing	921 (19.9%)

### PrEP use

The median duration of PrEP exposure within the period of observation (September 2019–December 2020) was 451 days (IQR 357–488), totaling 5132 py. More than one-half of PrEP users had started PrEP prior to September 2019 (*n* = 2466, 53.4%). Taking this into consideration, the median duration of PrEP exposure was 500 days (IQR 388–766), totaling 7353 py. Intermittent/on-demand PrEP was documented for 18.9%. In all, the average number of days of PrEP as prescribed divided by the number of days on PrEP (PrEP pill coverage) was 0.85 (SD = 0.23). Almost one-third (*n* = 1460, 31.6%) had a calculated PrEP pill coverage of less than 85%, which reflected both intermittent/on-demand PrEP use as well as a documented daily PrEP with lower adherence. The average PrEP pill coverage by mode of PrEP intake was 0.91 (SD = 0.16) for persons with PrEP prescribed for daily intake and 0.58 (SD = 0.28) for persons using PrEP intermittent/on-demand.

**Fig. 2:**
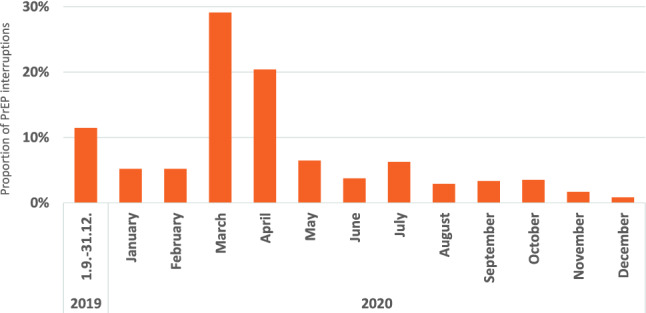
Proportion of PrEP interruptions over time from September 2019 to December 2020

**Fig. 3:**
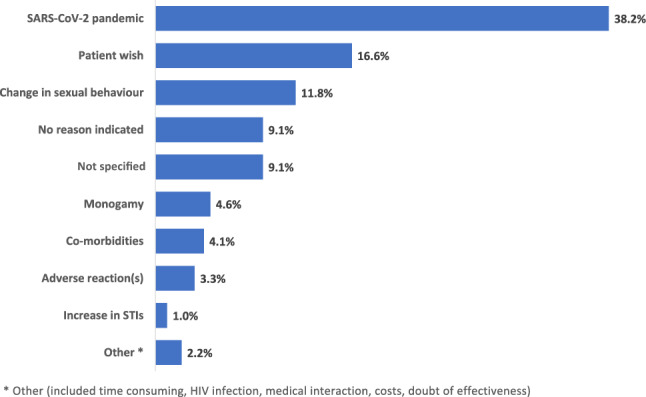
Primary reasons for discontinuing or interrupting PrEP. 905 reasons were documented for 725 individuals, combinations of reasons were categorized accordingly (multiple answers possible; proportion based on 905 reasons)

Ten percent of PrEP users had at least one PrEP interruption with a median duration of 93 days (IQR 58–148) with the first interruption after a median of 238 days (IQR 119–458) after PrEP start. PrEP discontinuations were documented for 13.2% after a median time of 275 days (IQR 142–478). Reasons for PrEP discontinuation or interruption were mainly SARS-CoV-2 related (38.2% of responses; 47.7% of persons); also, PrEP interruptions were highest in March and April 2020 (49.5% of all interruptions), at the time of the first COVID-19 lock-down (Figs. 2, 3). Whereas adverse reactions were rarely reported as the reason for PrEP discontinuation or interruption (3.3% of responses; 4.1% of persons) (Fig. 3).

Twelve percent (*n* = 555) did not have any event related to PrEP use (testing, diagnosis, treatment of STI, HIV, Hepatitis, or PrEP prescriptions) for at least two quarters before their last event, 84.5% (*n* = 469) of whom did not indicate PrEP discontinuation or interruption.

### HIV

During the observation period, four persons became diagnosed with HIV infection while on PrEP (MSM, age 26–33), accounting for 0.087% and an incidence rate of 0.078/100py (95% CI 0.029–0.208). HIV infections were diagnosed on day 32, 167, 270 and 295 after start of study observation, which corresponds to day 32, 167, 595 and 598 after PrEP start. For two individuals, PrEP on demand was documented, for the other two daily PrEP. The reason for infection in two cases was reported to be suboptimal adherence. In the third case, calculated PrEP pill coverage was 0.6 and resistance to FTC was observed. The fourth person, diagnosed on day 32 after PrEP initiation, reported condomless rectal intercourse a few days prior to PrEP start.

Three of these patients were also diagnosed with other STIs. Two of the individuals had presented with a syphilis infection in the previous 6 months before PrEP start and three had other STIs during the observation period (Table 2).

**Table 2:** Description of persons with HIV infections

Age	PrEP indication	PrEP use	Quotient Pills/days PrEP	PrEP start	PrEP Interruption (definition 4 weeks)	Last HIV negative test	HIV positive test	Days from PrEP start to HIV infection	Days from observation start to HIV infection	Reasons for HIV infection	HIV resistance	STI during PrEP use	STI before PrEP start
26	MSM	On demand	0.71	05.12.2019	No	11.03.2020	20.05.2020	167	167	PrEP on demand with poor adherence	No	No	No
27	MSM	Daily	1.82	06.01.2020	No	17.12.2019	07.02.2020	32	32	HIV risk before PrEP start	No	Gonorrhoe 2019q4	Unknown
33	MSM	On demand	0.75	01.02.2019	Yes 01.09.2019 –03.02.2020	27.01.2020	18.09.2020	595	270	PrEP on demand with poor adherence	Other resistance not TDF/FTC associated	Syphilis 2019q4	Syphilis
33	MSM	Daily	0.60	02.11.2018	No	23.01.2020	22.06.2020	598	295	Not documented	FTC resistance (M184IV)	Chlamydia 2020q1	Syphilis

### STI—chlamydia, gonorrhea, syphilis

Twenty percent of the study population were reported to have had an STI in the 6 months prior to PrEP start. The baseline STI prevalences were 7.01% (95% CI 6.05–8.09) for chlamydia, 6.62% (95% CI 5.68–7.66) for gonorrhea and 3.63% (95% CI 2.93–4.44) for syphilis.

**Fig. 4:**
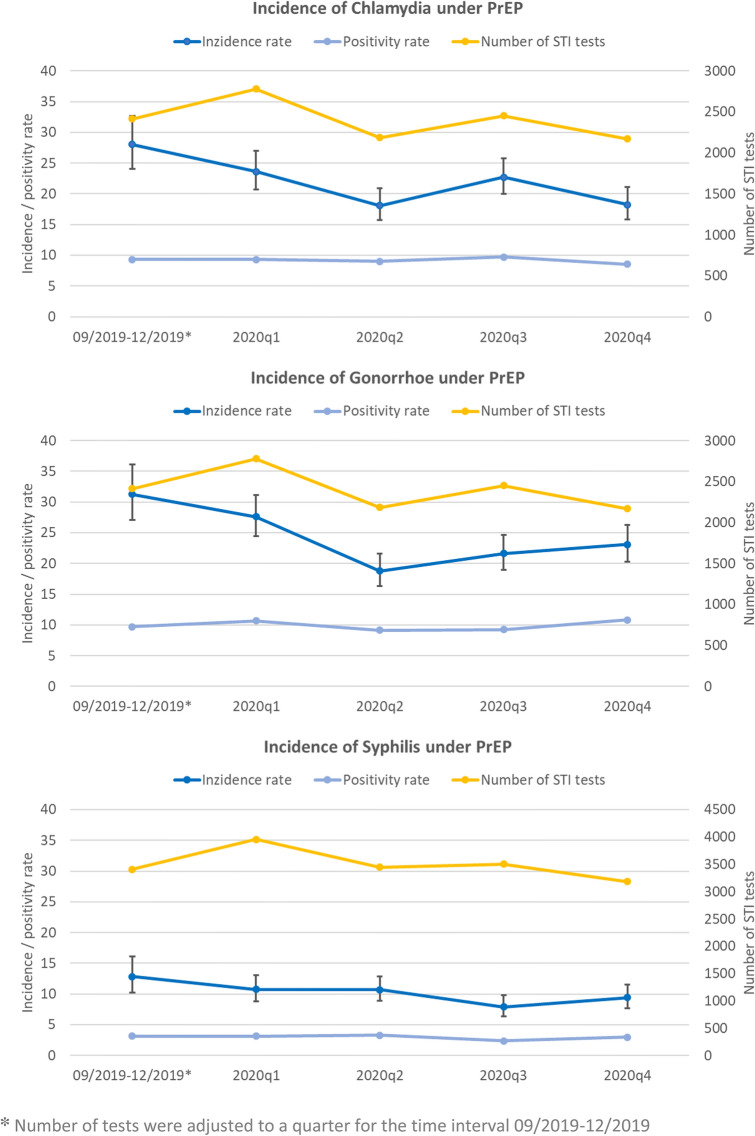
STI incidence rate, positivity rate and number of tests among German PrEP users over time from September 2019 to December 2020

Incidence rates during PrEP use in the observation period were 21.55/100py (95% CI 20.24–22.93) for chlamydia, 23.73/100py (95% CI 22.35–25.17) for gonorrhea and 10.08/100py (95% CI 9.18–11.03) for syphilis. The total incidence rate of any STI in our cohort was 55.36/100py (95% CI 53.24–57.54). The incidence per time period of any STI significantly decreased over time. Incidence rates over time are shown in Fig. 4 and Table 3 and were highest for all three infections in the first time interval (September–December 2019). Incidence rates already decreased in the first quarter of 2020. While the lowest incidence rates were seen in the second quarter of 2020 for both chlamydia and gonorrhea, it was lowest for syphilis in the third quarter. A rise in the incidence of gonorrhea and syphilis was again seen towards the fourth quarter of 2020 despite fewer tests. Overall, a significant reduction of 34.8% was found for chlamydia and of 26.1% for gonorrhea over the course of the observation period, as well as a reduction of 38.4% for syphilis from the first time interval through the third quarter of 2020. The total incidence of any STI showed a significant reduction of 29.6% over the course of the observation period.

**Table 3:** Incidence of STI infections on PrEP over time from September 2019 to December 2020

	09/2019–12/2019	2020q1	2020q2	2020q3	2020q4	Overall
Persons	1932	3842	4327	4226	4099	4620
Person years (PY) at risk	592.25	931.34	1054.07	1048.84	1017.91	4644.41
Positive results chlamydia	166	220	191	238	186	1101
Chlamydia incidence rate	28.03/100py	23.62/100py	18.12/100py	22.69/100py	18.27/100py	21.55/100py
95% CI	(24.07–32.63)	(20.7–26.96)	(15.72–20.88)	(19.98–25.77)	(15.83–21.1)	(20.24–22.93)
*P* value comparison*	Ref.	0.0957	< 0.0001	0.0364	0.0001	
Positive results gonorrhea	185	257	198	227	235	1102
Gonorrhea incidence rate	31.24/100py	27.59/100py	18.78/100py	21.64/100py	23.09/100py	23.73/100py
95% CI	(27.04–36.08)	(24.42–31.18)	(16.34–21.59)	(19.00–24.65)	(20.32–26.24)	(22.35–25.17)
*P* value comparison*	Ref.	0.1982	< 0.0001	0.0002	0.0020	
Positive results syphilis	76	100	113	83	96	468
Syphilis incidence rate	12.83/100py	10.74/100py	10.72/100py	7.91/100py	9.43/100py	10.08/100py
95% CI	(10.25–16.07)	(8.83–13.06)	(8.92–12.89)	(6.38–9.81)	(7.72–11.52)	(9.18–11.03)
*P* value comparison*	Ref.	0.2408	0.2248	0.0021	0.0440	
Positive results any STI	427	577	502	548	517	2571
Any STI incidence rate	72.10/100py	61.95/100py	47.62/100py	52.25/100py	50.79/100py	55.36/100py
95% CI	(65.42–79.27)	(57.00–67.22)	(43.55–51.98)	(47.96–56.81)	(46.51–55.36)	(53.24–57.54)
*P* value comparison*	Ref.	0.0174	< 0.0001	< 0.0001	0.0002	

*P value comparison for the incidence rates between quarters in reference to 09/2019–12/2019, *P* value obtained using the *χ*^2^-statistic [49]

Over one-third of the study participants (*n* = 1728, 37.4%) were reported to have had at least one STI in the observation period. During the study, 1203 tests for chlamydia were positive in 21.0% (971/4620 persons). For gonorrhea and syphilis, these were 1324 positive tests in 21.4% (987/4620 persons) and 602 positive tests in 8.9% (409/4620 persons), respectively. More than one infection with chlamydia, gonorrhea or syphilis were reported for 4.2% (194/4620 persons), 5.2% (238/4620 persons) and 1.6% (75/4620 persons), respectively. Sixty-seven individuals (1.5%) had infections with all three pathogens over the course of the observation.

Syphilis was tested for more frequently than chlamydia/gonorrhea (18 598 vs. 12 789 tests). (Fig. 2) The median number of tests per individual was three for chlamydia/gonorrhea (IQR 2–4, range 0–20) and four for syphilis (IQR 3–5, range 0–20). More than three-quarters had at least three tests for syphilis (*n* = 3630, 78.6%), while 43.6% (*n* = 2016) had at least three tests for chlamydia/gonorrhea. No testing for chlamydia/gonorrhea was documented for 22.6% (*n* = 1046), while no testing for syphilis was reported for 1.6% (*n* = 76). During the observation period, the lowest number of tests for chlamydia and gonorrhea were found in the second and fourth quarter of 2020 (*n* = 2186 and *n* = 2171, respectively) and for syphilis in the fourth quarter of 2020 (*n* = 3183).

The positivity rate remained relatively constant with a mean of 9.2%, 9.9% and 3.0% for chlamydia, gonorrhea and syphilis, respectively (Fig. 2). While 65.5% of syphilis diagnosis were detected by screening of asymptomatic individuals, these were 55.6% for chlamydia and 50.1% for gonorrhea. The proportion of screening tests of asymptomatic individuals in view of all tests was 84.1% (10 759/12 789) for chlamydia/gonorrhea and 94.9% (17 645/18 598) for syphilis.

The multivariable analysis of risk factors associated with chlamydia/gonorrhea infections among MSM showed a significantly higher risk for persons 16 to 29 years of age (OR 1.28, 95% CI 1.10–1.48), with PrEP start before statutory health insurance coverage (OR 1.23, 95% CI 1.06–1.43), with an STI history in the 6 months before PrEP start (OR 1.53, 95% CI 1.35–1.74) or an unknown STI history (OR 1.41, 95% CI 1.21–1.65), and with a higher number of symptom triggered tests for chlamydia/gonorrhea (OR 1.91, 95% CI 1.80–2.03). A significantly lower risk for infections with chlamydia/gonorrhea was found for persons 40–49 years of age (OR 0.77, 95% CI 0.68–0.88) or 50–59 years of age (OR 0.65, 95% CI 0.54–0.79), with intermittent/on-demand PrEP use (OR 0.78, 95% CI 0.66–0.91), and with a higher number of tests for chlamydia/gonorrhea when persons were asymptomatic (OR 0.93, 95% CI 0.88–0.97). A significantly lower risk for chlamydia/gonorrhea was also found for observations during the COVID-19 pandemic period (OR 0.77, 95% CI 0.67–0.89). (Supplement Table 1).

The analysis of risk factors associated with infections among MSM with syphilis showed a significantly higher risk for persons 50–59 years of age (OR 1.33, 95% CI 1.01–1.75), with an STI history in the 6 months before PrEP start (OR 3.32, 95% CI 2.66–4.14) or an unknown STI history (OR 1.75, 95% CI 1.29–2.39), and with a higher number of symptom triggered tests for syphilis (OR 2.16, 95% CI 1.91–2.43). A significantly lower risk for syphilis was found for observations during the COVID-19 pandemic period (OR 0.71, 95% CI 0.54–0.93). (Supplement Table 2).

### Hepatitis

In 3358 (72.7%) of individuals, hepatitis A immunity was reported, and 3664 (79.3%) had protection through antibodies against hepatitis B.

During the period of observation, two infections with hepatitis B were diagnosed on days 197 and 229 after start of observation, accounting for 0.043% of persons and an incidence rate of 0.04/100py (95% CI 0.01–0.16). Thirteen cases of hepatitis C were found at a median of 267 days (IQR 137–353) after start of observation in the study, and accounting for 0.28% of persons, a positivity rate of 0.30% (4273 tests) and an incidence rate of 0.25/100py (95% CI 0.15–0.44).

Additionally, one case of each, hepatitis A and B infection, and hepatitis C were diagnosed prior to or at the start of observation in the study (on days − 7, 0, and − 32 respectively).

## Discussion

To our knowledge, this is the first comprehensive description and analysis of real-life PrEP use in the largest available population of PrEP users in Germany. The cohort of PrEP users studied was predominantly male, MSM and in median 38 years of age. To estimate representativeness, we compared the demographic characteristics of our study population with health insurance data representing more than 50% of all persons with statutory health insurance in Germany and found them to be similar in gender, median age, PrEP use behavior, and regional distribution [21, 22]. To our knowledge, this is the only comparable source for Germany.

PrEP was found to be extremely effective in the prevention of HIV infection in this routine clinical setting. The observed incidence rate of 0.078/100py is comparable with and even lower than in other studies. The early iPrEX OLE study reported an HIV incidence of 1.83/100py and a strong protective effect with adequate drug levels. No HIV infection occurred in persons whose drug levels indicated an intake of four or more tablets per week [4]. More recently, the PROUD study found an incidence of 1.2/100py, the DISCOVER study an incidence of 0.34/100py in the TDF/FTC group and the IPERGAY study reported an incidence of 0.91/100py with on-demand TDF/FTC [23–25]. HIV incidence among PrEP users in implementation projects were closer to our findings with 0.16/100py in New South Wales (EPIC-NSW), 0.13/100py in England (PrEP Impact Trial), and 0.11/100py in France (ANRS PREVENIR) [26–28]. Differences in study populations and settings may partly explain differences in HIV incidence. In addition, our results are certainly influenced by the SARS-CoV-2 pandemic and the associated contact restrictions. However, a considerable rate of other sexually transmitted infections, shows that sexual contacts eventually took place during lockdown periods.

We found PrEP failure to be primarily linked to suboptimal adherence, which has been reported to be strongly correlated with efficacy [6, 29]. In our cohort, four persons were diagnosed with HIV while on PrEP. The person diagnosed on day 32 after PrEP initiation reported condomless rectal intercourse few days prior PrEP start and very likely was infected with HIV at baseline. In further two persons, acquisition of HIV infection was clearly linked to nonadherence. In one person failing on PrEP despite self-declared optimal adherence, resistance to FTC was documented. Yet, PrEP pill coverage for this person was calculated to be only 60%. And there were 5 months between the last negative and the first reactive HIV test. It, therefore, remains unclear whether this mutation was transmitted or selected for after transmission. Resistance to FTC is rare but well documented in persons with PrEP failure [6]. In addition, the presence of sexually transmitted infections may have facilitated HIV transmission in these persons [30].

The cohort of PrEP users studied was largely male, 99% MSM and 83% under the age of 50, which is similar to that in other implementation studies [26–28]. In Germany, PrEP appears to find minimal use outside the MSM community. This is supported by discussions with the community advisory board that took place within the scope of the EvE-PrEP project [31–33]. Other groups that may benefit from PrEP include persons within trans*/non-binary communities, sex workers, and individuals with certain migratory backgrounds [21, 22]. The SARS-CoV-2 pandemic has further contributed to a reduced focus on prevention including promoting PrEP especially in non-MSM groups [13]. Making PrEP available by eliminating barriers and increasing access for people at risk requires further attention.

PrEP use was most commonly prescribed for daily intake, with intermittent/on-demand PrEP use reported by less than one quarter. However, the calculated PrEP pill coverage suggests slightly higher intermittent/on-demand use, in addition to suboptimal adherence to documented daily PrEP. PrEP interruptions and discontinuations were reported for 10% and 13%. Since 12% of PrEP users did not have any event related to PrEP use in the last two time periods of observation, possible interruption or discontinuation may not have been reported. The actual rate may, therefore, be higher up to around 20%. Approximately one-half of the PrEP interruptions occurred in March and April 2020 at the time of the first COVID-19 lock-down, and the reasons for PrEP discontinuations or interruptions were mainly SARS-CoV-2 related. Adverse reactions played only a minimal role, as seen in other studies [6, 23]. This highlights the need for education to enable people interested in PrEP to make an informed, evidence-based decision as well as potential for a more wide-spread use of PrEP, considering that the fear of adverse reactions was one of the main reasons not to start PrEP in several surveys [21, 22].

One major concern of PrEP availability is the potential for increasing sexual risk taking and hence for acquisition of sexually transmitted infections. Previous publications on trends in STI for Europe showed decreasing HIV incidences but increasing incidences for early syphilis, gonorrhea and chlamydia since 2014 which was partly explained by higher PrEP use [34]. At baseline, 20% of our study population had a history of an STI in the previous 6 months which is associated with an increased risk of HIV infection [35]. During the observation period, 37% of the study participants were reported to have had at least one STI with a total incidence of any STI of 55.4/100py, indicating that these persons were sexually very active and that PrEP was adequately prescribed to persons at risk. The observed incidences of STIs vary among studies and are in the range of 72.2/100py [20] to 105.4/100py [27] depending on the population studied. In comparison, the incidence rates of STIs in our study were lower than in the before mentioned international studies [8, 20, 27]. Yet, the overall proportion of persons with an STI diagnosis in our study was only slightly lower than in other studies. In a German nationwide, cross-sectional study conducted in 2018 before the start of PrEP coverage by health insurances any STI with chlamydia, gonorrhea, Mycoplasma genitalium or Trichomonas vaginalis were documented for 25.0% of HIV-/PrEP- MSM and 40.3% in HIV-/PrEP + MSM [36]. In an Australian intervention study, 48% of participants were diagnosed with chlamydia, gonorrhea, or syphilis during a mean follow-up of 1.1 years [8]. While some evaluations showed an increase of STI infection rates during PrEP use over time [37], we found a significant decrease. This decrease is certainly in part attributable to the SARS-CoV-2 pandemic and the measures taken to control it as confirmed in the regression model. Yet, counselling and creating awareness as part of PrEP care may have also played a role.

The SARS-CoV-2 pandemic had a drastic influence on social behavior and sexuality. Yet sexual contacts in private settings took place with equal risk of HIV transmission. Sentis et al. found a 56% reduction in STIs during the March/April lockdown in Spain [38]. It was concluded that the decline probably was due to the effect of a combination of factors including change in sexual behavior as well as decreased availability of resources and decreased use of health care [38]. An analysis of notifiable STI case reports during the COVID-19 pandemic for 2020 in the US showed a clearly decreasing incidence of STIs during the strict lockdown time, but higher incidences thereafter for syphilis and gonorrhea [39].

Interestingly, in our study, rates over time also differed between STIs. Infections with chlamydia decreased with the lockdowns in the second and fourth quarters of 2020 and increased with social opening in the third quarter. After the initial decrease, infections with gonorrhea, on the other hand, showed a steady increase in both the third and the fourth quarters. Infections with Syphilis differed again, with their nadir in the third quarter, likely reflecting its longer incubation time [40, 41], followed by a slight rise in the fourth quarter of 2020. The incidence of Syphilis infections was slightly higher when compared to other studies [8, 27].

When evaluating infection rates, it is important to consider testing rates. Testing for chlamydia/gonorrhea decreased in the second and fourth quarters, which may reflect the pandemic lockdowns. Syphilis testing was higher throughout and decreased over the course of 2020. Interestingly although numbers of tests differed, the positivity rates remained relatively constant. Other investigators have reported decreased testing for other infections, reduced use of health care as well as a marked reduction of spread of communicable diseases, including STI and HIV, in times of social lockdown [42, 43]. In Germany, a drastic decline of notifications for most infectious diseases and pathogens was observed [44]. Of our study centers, 76% reported a decrease of requests for PrEP and 60% said PrEP check-ups were skipped during the first lockdown of the pandemic [13]. The COVID-19 pandemic and the measures taken to control it certainly influenced the results and outcomes of our study as confirmed in the regression model.

While almost one-half of chlamydia and gonorrhea infections were a result of screening of asymptomatic persons, this was the case for two-thirds of syphilis infections. The high rate of syphilis found solely by screening asymptomatic MSM highlights its importance in the prevention of transmission. The lower testing numbers for chlamydia/gonorrhea appear to reflect the differing approaches taken by physicians and the controversies surrounding testing and treating of asymptomatic individuals [14, 45].

The significantly higher risk of infection with chlamydia/gonorrhea for MSM aged 16–29 years and the significantly lower risk for MSM aged 40–59 years when compared to 30–39 years may reflect higher sexual activity and more partners in younger age groups. Unexpectedly, age 50–59 years was associated with an increased risk for syphilis. This finding is not supported by the national mandatory syphilis reports, which show the highest incidence for men between 30–39 years of age [46]. Further analysis in terms of study center location indicated that there may be an interrelated infection pattern that possibly explains this discrepancy.

A higher risk for chlamydia/gonorrhea was found in MSM with PrEP start before its coverage by statutory health care in September 2019. It likely reflects these individuals’ involvement in the sexually active MSM community. In addition, this indicates persistent HIV risk behavior, which goes along with infection risks of other STIs. The lower risk for chlamydia/gonorrhea seen with intermittent/on-demand PrEP use, on the other hand, may in turn reflect less frequent high-risk sexual contacts.

The lower risk for chlamydia/gonorrhea and syphilis during the COVID-19 pandemic period was likely caused by a number of factors as discussed above.

As expected, symptom triggered testing and a history of STI was associated with a higher risk for infections with chlamydia/gonorrhea and syphilis. The latter supports the importance of comprehensive history taking before PrEP initiation.

We found a high baseline protection against hepatitis A and B in approximately three-quarters of the cohort. Of note, immunity against hepatitis A may be in part self-reported and the actual percentage therefore lower supporting the call for serologic testing of immunity against hepatitis A prior to PrEP initiation. However, this is not covered by the German statutory health insurance nor recommended by the German/Austrian PrEP guidelines. Hepatitis B virus infection status on the other hand should routinely be determined prior to commencing PrEP according to the German/Austrian PrEP guideline due to the interaction between tenofovir and hepatitis B [47]. Accordingly, no infection with hepatitis A and only two with hepatitis B were diagnosed within the course of the study. However, thirteen cases of hepatitis C occurred, reflecting an ongoing epidemic of sexually transmitted hepatitis C among MSM, which in the past was almost exclusively seen among HIV infected MSM [48]. Additionally, at screening before PrEP initiation one case of each hepatitis A, B, and C were detected underlining the importance of a medically guided PrEP start.

Our study has several limitations: the evaluation period of sixteen months limits the ability to determine HIV and STI trends over time. Furthermore, detecting a hepatitis B/C infection can take several months after transmission. However, most of the participants were followed for a longer period with a median of 451 days within the observation period. The predefined four weeks for PrEP interruptions may have led to an underestimation of shorter PrEP interruptions. Also, the SARS-CoV-2 pandemic drastically affected social interaction and influenced the outcomes found in this study. Further investigation in a regular non-pandemic setting with a longer follow-up period would be valuable. The number of partners or sexual behaviors were not documented due to the retrospective chart review design of our study. Our population does not include all PrEP-users in Germany, nor all PrEP prescribing centers. For budgetary reasons participating centers could not document all PrEP users and were asked to include a random sample of their PrEP users into the study, however, no formal randomization process was implemented. This may have led to a selection bias. However, the fact that PrEP care is provided primarily by HIV-specialty care centers, many of which participated in this study with a German wide distribution and experience in clinical studies, lead us to assume that a representative sample was obtained. More than one-third of HIV-positive people receiving treatment in Germany and one-fourth of all PrEP users in Germany were cared for in the study centers [12, 14]. In addition, to estimate representativeness, the characteristics of PrEP users in NEPOS were compared with routine health insurance data analyzed in EvE-PrEP and were found to be similar.

## Conclusions

In Germany, HIV-PrEP with TDF/FTC is almost exclusively taken by MSM. We found a very low incidence of HIV infection in this cohort of PrEP users comparable to implementation projects in other countries. The main reason for PrEP failure was suboptimal adherence. We did not see an increase in infections with chlamydia, gonorrhea and syphilis; in fact, we saw a partial decrease in their incidence rates. The COVID-19 pandemic certainly influenced the findings of our study. Our results support the coverage of PrEP medication and care by statutory health insurance. Increased PrEP availability to people at risk for HIV infection through the elimination of barriers and improved access requires further attention. Further investigation and monitoring with a longer follow-up would be of value.

## Supplementary Information

Below is the link to the electronic supplementary material.

Supplementary file1 (DOCX 26 KB)

## Data Availability

The datasets generated and/or analyzed during the current study are not publicly available due to data protection and confidentiality but are available from the corresponding author on reasonable request.

## Abbreviations

CI: Confidence interval
COVID-19: Coronavirus disease 2019﻿
Dagnä e.V.: Deutsche Arbeitsgemeinschaft niedergelassener Ärzte in der Versorgung HIV-Infizierter e.V. (German)
DISCOVER: Study to evaluate the safety and efficacy of emtricitabine and tenofovir alafenamide (F/TAF) fixed-dose combination once daily for pre-exposure prophylaxis in men and transgender women who have sex with men and are at risk of HIV-1 infection
EPIC-NSW: Expanded PrEP implementation in communities in New South Wales
EvE-PrEP: Evaluation der Einführung der HIV-Präexpositionsprophylaxe als Leistung der Gesetzlichen Krankenversicherung (German)
HIV: Human immunodeficiency virus
IPERGAY: Intervention préventive de l’exposition aux risques avec et pour les GAYs (French)
iPrEX OLE: “Iniciativa Profilaxis Pre-Exposicién” (Spanish), open label extension
IQR: Interquartile range
MSM: Men who have sex with men
NEPOS: National Evaluation of PrEP Outcomes and STIs
OR: Odds Ratio
PrEP: Pre-exposure prophylaxis
PREVENIR: Prevention of HIV in "Île-de-France"
PROUD: Pre-exposure option for reducing HIV in the United Kingdom
py: Person years
RKI: Robert Koch Institute
SARS-CoV-2: Severe Acute Respiratory Syndrome Coronavirus-2
STI: Sexually transmitted infection
TDF/FTC: Tenofovir disoproxil fumarate and emtricitabine
