# Supplementary material for: Low incidence of HIV infection and decreasing incidence of sexually transmitted infections among PrEP users in 2020 in Germany
Source: Infection. 2022 Sep 27;51(3):665–78. doi: 10.1007/s15010-022-01919-3 (PMC9514183; doi:10.1007/s15010-022-01919-3)
Supplement: Supplementary file 1 — Supplementary file1 (DOCX 26 KB) [file 15010_2022_1919_MOESM1_ESM.docx]

**Supplement table 1**

**Mixed-effects logistic regression** Number of obs = 10,758

Group variable: ANR Number of groups = 47

Obs per group:

min = 14

avg = 228.9

max = 827

Integration points = 7 Wald chi2(18) = 682.09

Log likelihood = -4749.1181 Prob > chi2 = 0.0000

| **Chlamydia/Gonorrhea infection**  **Total N=10758 events (among MSM)** | **Odds ratio** | **P>z** | **[95% CI]** | |
| --- | --- | --- | --- | --- |
| Age |  |  |  |  |
| 30-39 | Ref. |  |  |  |
| **16-29** | **1.28** | **0.001** | **1.10** | **1.48** |
| **40-49** | **0.77** | **0.000** | **0.68** | **0.88** |
| **50-59** | **0.65** | **0.000** | **0.54** | **0.79** |
| 60-69 | 0.80 | 0.250 | 0.54 | 1.17 |
| PrEP use |  |  |  |  |
| Daily | Ref. |  |  |  |
| **On-demand** | **0.78** | **0.002** | **0.66** | **0.91** |
| PrEP interruption |  |  |  |  |
| No | Ref. |  |  |  |
| Yes | 0.83 | 0.206 | 0.62 | 1.11 |
| PrEP discontinuation |  |  |  |  |
| No | Ref. |  |  |  |
| Yes | 0.83 | 0.072 | 0.68 | 1.02 |
| PrEP duration (days) | 1.00 | 0.183 | 1.00 | 1.00 |
| PrEPStartBefore |  |  |  |  |
| Nein | Ref. |  |  |  |
| **Ja** | **1.23** | **0.006** | **1.06** | **1.43** |
| Hepatitis infection |  |  |  |  |
| No | Ref. |  |  |  |
| Yes | 1.13 | 0.908 | 0.14 | 9.18 |
| HIV infection |  |  |  |  |
| No | Ref. |  |  |  |
| Yes | 1.70 | 0.555 | 0.29 | 9.85 |
| Syphilis infection |  |  |  |  |
| No | Ref. |  |  |  |
| Yes | 1.19 | 0.203 | 0.91 | 1.55 |
| STI history before PrEP |  |  |  |  |
| No | Ref. |  |  |  |
| **Yes** | **1.53** | **0.000** | **1.35** | **1.74** |
| **Unknown/missing** | **1.41** | **0.000** | **1.21** | **1.65** |
| **Number of asymptomatic Chlamydia/Gonorrhea tests** | **0.93** | **0.002** | **0.88** | **0.97** |
| **Number of symptomatic Chlamydia/Gonorrhea tests** | **1.91** | **0.000** | **1.80** | **2.03** |
| Covid-19 pandemic |  |  |  |  |
| No (09/2019-03/2020) | Ref. |  |  |  |
| **Yes (03/2020-12/2020)** | **0.77** | **0.000** | **0.67** | **0.89** |
| _cons | 0.20 | 0.000 | 0.17 | 0.25 |
| Note: _cons estimates baseline odds (conditional on zero random effects). | | | | |
| **Random-effects parameters** | **Estimate** | **Std. err.** | **[95% CI]** | |
| ANR: Identity |  |  |  |  |
| sd(_cons) | 0.43 | 0.07 | 0.32 | 0.58 |
| LR test vs. logistic model: chibar2(01) = 107.77 Prob >= chibar2 = 0.0000 | | | | |

**Supplement table 2**

**Mixed-effects logistic regression** Number of obs = 16,289

Group variable: ANR Number of groups = 47

Obs per group:

min = 22

avg = 346.6

max = 935

Integration points = 7 Wald chi2(18) = 339.59

Log likelihood = -1971.451 Prob > chi2 = 0.0000

| **Syphilis infection Total N=16289 events (among MSM)** | **Odds ratio** | **P>z** | **[95% CI]** | |
| --- | --- | --- | --- | --- |
| Age |  |  |  |  |
| 30-39 | Ref. |  |  |  |
| 16-29 | 0.87 | 0.354 | 0.64 | 1.17 |
| 40-49 | 0.92 | 0.473 | 0.73 | 1.16 |
| **50-59** | **1.33** | **0.044** | **1.01** | **1.75** |
| 60-69 | 1.44 | 0.177 | 0.85 | 2.44 |
| PrEP use |  |  |  |  |
| Daily | Ref. |  |  |  |
| On-demand | 0.83 | 0.208 | 0.62 | 1.11 |
| PrEP interruption |  |  |  |  |
| No | Ref. |  |  |  |
| Yes | 0.93 | 0.806 | 0.51 | 1.69 |
| PrEP discontinuation |  |  |  |  |
| No | Ref. |  |  |  |
| Yes | 0.65 | 0.041 | 0.43 | 0.98 |
| PrEP duration (days) | 1.00 | 0.623 | 1.00 | 1.00 |
| PrEPStartBefore |  |  |  |  |
| Nein | Ref. |  |  |  |
| Ja | 1.11 | 0.453 | 0.85 | 1.46 |
| Hepatitis infection |  |  |  |  |
| No | Ref. |  |  |  |
| Yes | 2.19 | 0.472 | 0.26 | 18.58 |
| HIV infection |  |  |  |  |
| No | Ref. |  |  |  |
| Yes | 3.12 | 0.312 | 0.34 | 28.35 |
| Chlamydia infection |  |  |  |  |
| No | Ref. |  |  |  |
| Yes | 1.30 | 0.095 | 0.96 | 1.77 |
| Gonorrhea infection |  |  |  |  |
| No | Ref. |  |  |  |
| Yes | 0.85 | 0.347 | 0.60 | 1.19 |
| STI history before PrEP |  |  |  |  |
| No | Ref. |  |  |  |
| **Yes** | **3.32** | **0.000** | **2.66** | **4.14** |
| **Unknown/missing** | **1.75** | **0.000** | **1.29** | **2.39** |
| Number of asymptomatic Syphilis tests | 0.97 | 0.495 | 0.88 | 1.06 |
| **Number of symptomatic Syphilis tests** | **2.16** | **0.000** | **1.91** | **2.43** |
| Covid-19 pandemic |  |  |  |  |
| No (09/2019-03/2020) | Ref. |  |  |  |
| **Yes (03/2020-12/2020)** | **0.71** | **0.015** | **0.54** | **0.93** |
| _cons | 0.02 | 0.000 | 0.01 | 0.03 |
| Note: _cons estimates baseline odds (conditional on zero random effects). | | | | |
| **Random-effects parameters** | **Estimate** | **Std. err.** | **[95% CI]** | |
| ANR: Identity |  |  |  |  |
| sd(_cons) | 0.90 | 0.12 | 0.69 | 1.16 |
| LR test vs. logistic model: chibar2(01) = 417.04 Prob >= chibar2 = 0.0000 | | | | |
